# Conference at Birmingham

**Published:** 1912-10-05

**Authors:** 


### THE BRITISH HOSPITALS ASSOCIATION.

### CONFERENCE AT BIRMINGHAM.

### Full Report of the Proceedings and Discussions.

Resuming at 2.30, the Conference listened to papers by Dr. Nathan Raw (Mill Road Infirmary, Liverpool) on "The Probable Effect of the Insurance Act on Voluntary Hospitals and other Institutions," and by Mr. T. Basil Rhodes (Secretary and House Governor, North Staffordshire Infirmary, Stoke-on-Trent) on "The Voluntary Hospitals and the National Insurance Act," which for the sake of convenience were both read before any discussion took place, and the former of which we print. Owing to pressure of space Mr. Kemp's paper on "The Training and Work of the Almoner" and the discussion on it has had to be omitted.

### The Probable Effect of the Insurance Act upon Voluntary Hospitals and other Institutions.

There can be little doubt that the passing of the Insurance Act will have a far-reaching and serious effect on the working and maintenance of our present voluntary hospitals. It only remains for us to discuss and carefully consider the best way to counteract the effect of the Act upon the hospitals, without in any way endangering their efficiency. By far the most important factor for the maintenance of our voluntary hospitals is money, and without a liberal supply of money their very existence is threatened. The two great systems in vogue at present in Great Britain for the treatment of disease are the voluntary hospitals and Poor-Law institutions. The voluntary-hospital system is essentially founded on charity; the Poor-Law institutions are based on destitution. The democrats of this country are just as much opposed to the accepting of charity at the present day as they are to receive help from the Poor Law. It is not that the services in each class are not good, but it is the spirit in which they are offered which causes irritation. Now it must be admitted that the original functions of these two great systems for the relief of human suffering have to a great extent changed; a large proportion of those who use our excellent voluntary hospitals do not do so in any spirit of charity, and a large number of people who use the Poor-Law institutions, more especially the well-equipped and up-to-date Poor-Law infirmaries, are not by any means in a state of destitution. The fact is that both these great systems have modified their original cast-iron principles to suit the needs of the present time, and it is entirely on this account that further alterations in their constitution will have to take place to bring them into line with a great administrative measure like the present Insurance Act. The health of a nation is much too vital a matter to be left to voluntary effort and to the chance flow of legacies and charitable subscriptions.

Those who make use of voluntary hospitals should pay for services received according as their circumstances will permit, whilst those who render such services should also be paid, not, I am afraid, in any ratio to their value, but in the shape of a substantial honorarium. The committees of voluntary hospitals are engaged all the year round in a continuous fight to obtain money, and there can be no doubt that in many institutions efficiency is, and has to be, to some extent sacrificed on that account. Another result is that the nursing staff is compelled to work long hours with a totally inadequate remuneration for their trained services.

My experience in the administration of hospitals during the last twenty years has profoundly impressed me with the fact that a great change in our hospital system is inevitable. Our voluntary hospitals and our system of nursing have been the admiration and envy of the world, and so long as their work was confined within ordinary limits all was well, but the work has become too onerous and the number of persons who are now anxiously clamouring for hospital treatment is so enormous, that it is impossible to find the money requisite adequately to treat this army of sick people. The fact is, that the whole undertaking must be worked on sound business lines, and the treatment of disease must be conducted in the same way as any other municipal enterprise.

Another point which has always impressed me is the inequality of treatment under the present system. Those who are fortunate enough to gain admission to a voluntary hospital receive all necessary treatment, and may be sent to a convalescent home to recuperate without any question being asked as to payment, whilst those (in the same class of society) who are taken to Poor-Law institutions are carefully scrutinised and made to pay, if possible, the full cost of their maintenance.

Of the two systems the treatment under the Poor Law is undoubtedly the most equitable, and experience has shown that even the poor make no objection to pay for their treatment, and in a great many cases prefer to pay something. The Insurance Act will undoubtedly hasten on some such scheme with regard to voluntary hospitals, though I would be sorry to see all hospitals supported by rates. Yet I am convinced that a graduated system of patients' payments would do more than anything else to obviate that contingency. It might be necessary to receive subsidies from the insurance funds or from the municipality to meet deficiencies each year.

Under the Insurance Act two outstanding facts are before us: (1) No provision is made for the hospital treatment of insured persons; (2) no insured person may be treated by the Poor-Law authorities. I estimate that when the Insurance Act is in full swing 30,000 beds will be required to deal with the illnesses of 15,000,000 insured persons. It was a grave defect of the Act that a carefully considered plan had not been prepared when it was framed, but perhaps this defect is only of a temporary character, and that when the medical benefits are brought into force a proper arrangement will be made for hospital treatment. Undoubtedly more beds will be required in hospitals than are at present at the disposal of the working classes, as it is most undesirable that acute diseases should be treated in the homes of the insured, whilst surgical operations can only be properly conducted and nursed in a well-equipped hospital. In my opinion, in the course of a few years, when the funds have accumulated, the Insurance Committees will provide their own hospitals for the sole use of insured persons, with a properly paid professional staff, on the lines on which many of the German hospitals are conducted, and which work so smoothly. I have visited many of these institutions in Germany, built and equipped out of the surplus insurance funds, and they are certainly as up to date and as perfect as money and brains can make them.

With regard to the position of the Poor Law, there can be no doubt that the Insurance Act has been framed with the deliberate intention of avoiding it altogether in the treatment of insured persons. This will mean that the thousands of workers, including labourers, domestic servants, and others earning low wages, will no longer be allowed to be treated in the Poor-Law infirmaries, and that accommodation will have to be found for them in other hospitals, and, moreover, there is nothing to prevent the present inmates of the Poor-Law institutions from becoming insured persons (if they are able to work) and joining as Post-Office contributors. This section will include some thousands of persons suffering from tuberculosis, who are at present excellently treated under the Poor Law.

This short paper will not permit me to enter into detail as to how these workers are to be treated, or as to how the accommodation is to be provided, but I sincerely hope that the voluntary hospitals will all be prepared to offer treatment to insured persons on terms to be arranged, and so obviate the necessity of special hospitals being provided. The voluntary hospitals may adopt one of several courses :

(*a*) Continue as voluntary institutions and disregard the Act.

(*b*) Continue as voluntary institutions and accept all insured persons at a fixed rate per day, to be paid by Insurance Committees.

(*c*) Cease to be voluntary institutions and have a fixed scale of charges—first, second, and third class; the beds to be open to all citizens (the insured persons would be third class and pay something like 2s. 6d. per day).

(*d*) Hand hospital over bodily to the Insurance Committee as a State Insurance Hospital, as will probably be done in the case of separate Poor-Law infirmaries, and changing their name.

For my own part, I prefer the change under heading (*c*), as being a logical and equitable solution of the changing times. It would give every resident the right to use the hospital according to his means, and those who were unable to pay would be provided for out of public funds on the model of Austria and other countries. All these institutions should be available for education and research, and the chief physicians and surgeons recognised as teachers by the Universities in all hospitals containing over 200 beds.

### The Discussion.

Mr. Conrad W. Thies, Royal Free Hospital, London:—

I have been struck by the fact that the two papers we have heard were both from gentlemen who had had experience in large manufacturing districts—that is to say, districts where there are a large number of the working-class population, and where the contributions are on a very large scale as compared with what any hospital in London could possibly show. There is no doubt that the support given by the working-class people to these large provincial hospitals has been large and a great credit to the class. But the conditions in London are very different. The contributions received direct from the working class by the hospitals—about a hundred and thirty in number—at the present time are so small that they are hardly worth consideration. The experience of the whole world outside of this country has led to the taking up of this question of the health of the people by the community taking the responsibility of providing hospital accommodation in some form or another. You may look abroad in every direction—throughout the whole of Europe, in America, in our Colonies—and you will find what I say is correct. In this country we have got the two systems gradually working up together. What is required to-day, and is supplied in every other country, is provision for the class of person above the poor. It seems to me utterly unreasonable that such splendid arrangements should be made for the treatment of a comparatively small class of the population, while the working people, upon whom the real prosperity and growth of the country depend, should not have adequate provision made for them when they can pay for it. In the German hospital there are four classes. There are the very poor, who are paid for by the district they come from at about 1s. 6d. per day. The class above that is the insured persons, who really and truly are the largest proportion of the patients in the hospital. They are paid for under the Insurance scheme. The third class is the upper or better-middle class, who are able to pay, and are required to pay at a certain definite rate. Above all is the well-to-do class, which pays. The result of that is that it destroys the nursing-home system. In London to-day we have between 300 to 400 nursing homes. Many are excellent, but a very large number are absolutely unsuitable, and are carried on simply with the object of making money and under no inspection whatever. I think the inevitable result must be that some scheme by which persons of all classes must have the right to have the best treatment possible to be obtained by each paying according to his means, those unable to pay being paid for by the districts they come from, not as under the Poor Law, but by some contribution from the district, or by contributions from the Insurance Fund. I have not the slightest doubt in my own mind that Mr. Lloyd George has had that definitely in his mind. He meant that in some way or other the voluntary hospitals would have to be incorporated into some scheme like the German or Swiss system and contributions from the State given to their support.

Mr. J. N. Smethurst, Warrington Infirmary:—

I can hardly agree with the suggested differentiation in the payment made by people who come to the hospital. In Warrington we have a system by which when a patient comes to the hospital his name is entered in the books with particulars obtained from the patient or his friends as to how much or how little can be paid. We do consider that if they can pay us something towards the cost they ought to do so, but that is a very different thing from first, second, and third class patients. Dr. Rhodes' suggestion is the only one that I have heard or read which is a practical hint as to how to cope with our difficulties, and that is the mention he makes at the end of the paper in dealing with a supposed deficiency at the end of the financial year. I am very much afraid that it would not be possible to persuade this or any other Government to foot the bill of deficiencies that would arise. I rather imagine that there would then be a tendency among the public generally not to subscribe. I do agree with him that the medical profession would be wrongly advised not to come to terms. We hear a great deal as to what is a fair thing between the medical men and the Insurance Commissioners as a whole; we shall have to consider what is a fair thing when the medical men say they can no longer work in an honorary capacity. My men at home told me I must go to the local Health Insurance Committee and tell them I must have so much per head for an insured person, and that I must give them something for their part. I said, "How much do you want for your part?" And they said, "We shall not squabble about that." You may not squabble, but you would like to know. So I asked, but I am not in a position to be able to tell you how much they want. They said, "You go to the Conference, and when you come back tell us what other people want." (Laughter.) The medical men should say how much they want. Then we should be in a position to go to the Health Authority and say, "Our medical men want so much and our costs are so much," and so long as the hospital authorities run the hospitals so efficiently and at a minimum cost they are entitled to be refunded.

Mr. Richard Kershaw, Secretary of the Central London Throat, Nose, and Ear Hospital:—

I firmly believe that whether recent legislation had taken place or not, the hospital system in this country is slowly moving in the direction of either State aid or perhaps State control. If you get State control, then I think what will follow will be a nationalisation of the entire medical service. The hospitals twenty years ago were the most conservative institutions in the country; to-day they are the most democratic. They are democratic, as has been explained in the paper of Mr. Rhodes, and the way in which the working-men should support these institutions. When I first entered the hospital world there was no such thing as Hospital Saturday or the workmen's contributions which you have to-day, but with that change you have also got a change in the constitution of the hospital. There was certainly not the class of patient attending the hospitals there is to-day. But I do not want to be understood to say that those patients who attend to-day ought not to be there. They ought to be there. But what concerns hospital officers most is how in future are we to collect the money? What I should like to see is a partnership, co-operation. I want to see further co-operation. You have got it with your workmen's contributions. I want to see further co-operation between the patient, the State, and the medical practitioner. Take the income of my hospital; how is it derived? We collect £4,000 a year; we spend a little less. Fifty per cent. of our income is derived from patients' contributions. You may say, "Yes; you take so much money from your patients who ought to be in the consulting rooms of the medical practitioners," but 40 per cent. of those paying people come from the medical practitioner to the hospital. That is the co-operation that I should like to see further extended. A few years ago one of our central organisations for the collection and distribution of money appealed for £100,000 in order that the hospitals of London might be freed from debt. That £100,000 has been collected and £50,000 beyond it every year, and yet we are more in debt than ever. When I spoke of co-operation between the patients, the medical practitoner, and the State I did not intend to omit the present benevolent subscriber, a system which is the envy of this country and the admiration of every other. A few more figures concerning the work of the hospital with which I am connected may be of interest. I should like to tell you that we treat 12,000 out-patients every year, and when I first went to the hospital 54 per cent. of them were admitted entirely free, either with a subscriber's letter or free, sickness being the only passport needed. The position to-day is entirely reversed, 54 per cent. paying and the balance being admitted free. There, again, you have got a democratic position of the hospital, which I for one so much desire to see continued.

Mr. J. Courtenay Buchanan, Metropolitan Hospital, London:—

The only comment I have to make on the papers we have heard read is on that of Dr. Nathan Raw. He has drawn attention in the second paragraph to what he calls the totally inadequate remuneration of the nurses for their trained services. Dr. Raw has probably forgotten that in the average voluntary hospital in London, and, I have no doubt, in the provinces as well, the proportion of nurses per patient is very much greater than in the hospitals supported by the Poor Law and out of the rates. Various suggestions, however, for meeting our difficulties have been laid before the Conference, and measures will be taken to further any decisions. The position is now very complex. Nevertheless, the present National Insurance Act starts a new era in the history of the voluntary hospitals. Changes in hospital administration must soon come about. In my judgment there will be two direct effects—one on the class and the method of admission of cases, and the other on the method by which the hospitals obtain their income. One of the anomalies of the present hospital system is that in this country we provide in the voluntary hospitals for very poor, sometimes *bona-fide* Poor-Law cases, treatment such as none but the exceedingly wealthy could obtain in their own homes. The intermediate class have little, if any, care or provision made for them. I hope that in the near future this anomaly may be removed. I welcome the frequent recurrence to-day of the words "co-ordination" and "co-operation." The proper functions of the hospitals are: (1) the treatment of the poor; (2) medical and nursing education; and (3) scientific research.

The Rev. Henry Stuart, North Staffordshire Infirmary, Stoke-on-Trent:—

Dr. Raw suggests that from his point of view the most desirable thing is that they should cease to be voluntary hospitals. Nevertheless, the aim should be the subsidising of the voluntary hospital rather than its supercession. We suffer considerably, I believe, in connection with education and with the Poor Law from being too much managed by officials and experts. There is a second question, in some respects more important for us just now. What about this period of transition in which we find ourselves? I want to put in a very strong plea that at the present time we should hold our heads up and believe that things will be better than we fear. One point in Mr. Rhodes' paper with which I cannot agree is his last suggestion that the State should make up our deficiencies. There are many ways in which we could spend it needfully if we were quite sure it was going to be made up at the end of the year, which might not be said to be really required, although perhaps justified. I hope the State may be induced in this time of transition to come to our aid rather by a scheme of helping us in the proportion in which we help ourselves, as in New Zealand, where, I believe, the State provides 25s. for every guinea privately subscribed. It has been found possible to provide extra grants in connection with education for necessitous areas, and it seems to me not impossible that some similar arrangement might be made in connection with hospital work. One thing that interested me very much in New Zealand in connection with the hospitals was this: It seemed to me that State aid being a very large proportion of the amount contributed for the maintenance of the hospitals, it would be natural to expect that the medical men were paid for their services in the hospitals, but as a matter of fact that does not obtain. I was told on very good authority by one of the best known surgeons in Auckland that there is no difficulty in getting the most efficient physicians and surgeons to serve in the hospitals in New Zealand, though they are doing their work just as much in an honorary way as at home.

Mr. Stewart Johnson, Children's Hospital, Great Ormond Street, London:—

I think it has been taken for granted by the previous speakers that the subscriptions of the hospitals are going to fall off, and that having to make them up in some way we shall have to receive a subsidy in some form or other from the State. Various suggestions have been made. In Australia and New Zealand I believe the subsidy in proportion to the subscriptions is not found to work at all well. The lump sum method is open to all sorts of corruption and lobbying and many other evils, while to assess the sum would require the maximum amount of inspection possible. Who is to give the subsidies—whether it is to be the Insurance Committees, or the municipalities, or whether it is to be what one calls the State? I suppose that is a kind of national grant in aid. As to the effect on hospitals of the working-men having more say in the matter than previously, we have got to "educate our masters." We have got to educate our patients and the class from which the patients are drawn.

Mr. Harold Wigg, Secretary of the Walsall and District Hospital:—

I endorse the optimistic opinion expressed by Dr. Rhodes as regards the possibility of maintaining the voluntary support which the hospitals require. It would be a very good thing if groups of hospitals could form joint boards for the purpose of obtaining voluntary support. Such groups would be in a very much stronger position to get support than the hospitals now. In towns such as ours it is so difficult to overcome old prejudices in order to institute anything which may be beneficial in the administration of the institution.

Mr. J. W. Cooke, Honorary Secretary Albert Infirmary, Winsford, Cheshire:—

I have every faith myself that subscriptions will continue just the same. But it is absolutely impossible for the hospitals to get on unless they receive the support of the medical profession. As a rule we keep his Majesty out of everything, but this is not a political question. If his Majesty could bring about an arrangement between the hospitals and the medical profession, why should not his Majesty undertake the duty, assisted by the Archbishop of Canterbury and the Lord Mayor of London? (Laughter.) You may feel amused, but unless agreement is reached there is going to be a good deal more strife, which probably may pay the lawyers better than the doctors.

Mr. Mansell, Chairman of the Salop Infirmary, Shrewsbury:—

So far as our hospital is concerned, we could not deal with all the people who would have to be dealt with under the Insurance Act. I have always felt that the rich man and the poor man were well looked after; but the man of moderate means, with perhaps a wife and child dependent upon him, who had unfortunately to be operated upon, is often ruined.

Mr. E. J. Thomas, Ear and Throat Hospital, Birmingham:—

I am one of the representatives of the Hospital Saturday Fund on the Ear and Throat Hospital, and have been a representative on the Birmingham Dispensary. I have tried to get in all the various circles in which I move some idea of what the effect will be of the National Insurance Act on the hospital, and frankly I have failed. There seems to me no reason why the Insurance Commissioners should not pay for the maintenance of the hospitals. From the working-man's point of view I feel sure that we shall lose subscriptions. We cannot find out what will be the effect on the Hospital Saturday Fund. I think we shall muddle through.

Mr. F. W. Francis, Member of the Committee of the Warneford Hospital, Leamington:—

I would like to impress upon the members of the medical profession not to be too hurried in carrying out their resolve not to attend an insured person after January 1. It is absolutely impossible for the Insurance Committees to find the money for hospital treatment at that time. The funds of the Insurance Committee are allocated by a calculation made by accountants, and the only margin, I believe, on the funds available for medical benefit is something like 10 per cent., and that margin is the margin out of which the Act allows us to make contributions to voluntary hospitals. The medical benefit under the Act does not mean hospital treatment at all, but domiciliary treatment, so that there is no margin for any beyond small subscriptions to hospitals, and thus I am afraid the fund available will be swallowed up by the increased demands of the medical profession, until the County Council, or Town Council, or the Government place more at the disposal of the Insurance Committee. Then I do not understand that thousands of insured persons will any longer be allowed to be treated in the Poor-Law infirmaries. There is no duty in the Act at all for Insurance Committees to provide hospital treatment. All we can do if we have funds is to make a subscription to voluntary hospitals. Of course, sanatoria treatment is different, but there is no provision for us to use sanatoria benefit for anything but tuberculosis. Then it seems to me that it would be quite possible for Boards of Guardians to continue to treat the same class of people in the Poor-Law infirmaries as they do at present.

Dr. Raw: Not if they are insured.

Mr. Francis: I should like to know why. I do not see anything in the Act to prevent their continuing as before.

Mr. Kay, Bolton Infirmary:—

We have a splendid Hospital Saturday Committee, and they intended to make the annual amount £5,000 this year, but instead of reaching previous totals it has receded, and all on account of stamps. (Laughter.) The impression throughout Lancashire is that they are paying stamp duty and that there is no need to support the infirmary. Our committee are trying to convert the people, but I do not know how we shall go on.

Mr. Spruce, Swindon:—

Mr. Rhodes has said that he does not think we shall get a blank cheque from Mr. Lloyd George to cover deficiencies; neither do I. At the same time I think the workers will do their part. I should like to ask Mr. Kemp what he considers a fair maximum wage for the workers to put them outside the scope of the scheme.

### The Readers' Replies.

Dr. Nathan Raw : In my opinion the great question which has forced the issue regarding the voluntary hospitals has been the progress which has been made in the treatment of the sick under the Poor Law, and I think we do not appreciate the enormous number of the poorer classes of this country who are treated efficiently and well under the Poor Law at the present time. When the Poor Law was first introduced into this country there was no intention that it should ever deal with sick persons. It was a case of dealing with destitution, but evolution has solved that. The result was that the Poor Law set up fine hospitals and infirmaries, and they are doing work done originally by the voluntary hospitals. The result of that is this, that the community as a whole having paid for the maintenance of the Poor-Law infirmaries through the rates did not see the necessity of subscribing to the voluntary hospitals. They say that the really deserving poor are provided for by the Poor Law and that there is no necessity to subscribe for their maintenance in the voluntary hospitals. That is the reason for the unrest in the voluntary hospitals during the last fifteen years. It has been evident for the last ten years that some change would have to take place with regard to the maintenance of the voluntary hospitals, that they could no longer be continued as purely voluntary institutions. Now we have the Insurance Act, which is going to compel insurance from fifteen million people. The voluntary hospitals cannot possibly cope with the enormous amount of sickness which must be treated in hospitals, and it is also obvious that the honorary staffs, the medical and surgical men attached to them, could not possibly be expected to treat insured persons on the same lines as poor, deserving people. Therefore some arrangement will have to be made, not only for the proper payment of medical men attending to insured persons, but also for the maintenance of those insured persons in our voluntary institutions. In my opinion it will be a compromise; that the voluntary institutions will remain to some extent voluntary, but that they will have to be subsidised either by the Insurance Committees or the State itself. I should be very sorry to see the magnificent voluntary institutions interfered with, but it is quite impossible to find the money to maintain them in the present efficiency, and therefore we come down to the bedrock of the whole problem, and that is who is going to find the money. In my judgment the insured persons who use the hospitals must fairly be asked to contribute for their maintenance, either directly by themselves, or through their societies, and that would be received by the committees of the voluntary institutions as a means of keeping the institutions above water. Mr. Smethurst raised the point as to how much money the doctors ought to have. It is notorious that doctors care little about money—(laughter)—and therefore no difficulties will arise so long as a fair and reasonable amount is offered.

Mr. Rhodes : With regard to my suggestion about State aid, I beg you to consider it. My suggestion is not quite the payment of a lump sum. It is the making up of a deficit, which is a rather different matter, and I think it might be used as a means to get more subscriptions rather than to allow subscriptions to drop down.

The Conference then adjourned until the following (Friday) morning, and a reception was held by the Lord Mayor.

### SECOND DAY'S PROCEEDINGS.

The proceedings were resumed on Friday morning at ten o'clock. Mr. J. B. Clarke again presided.

The Hon. Secretary (Mr. Howard Collins) announced that the attendance at Thursday's Conference numbered 126 in the morning and 98 in the afternoon.

Mr. J. Danvers Power, M.V.O., Member of Council and King Edward's Hospital Fund for London, then read his paper on

### Hospital Management Considered with Reference to Responsibility.

The managers of an English voluntary hospital are, as everybody knows, responsible to no public authority, so long as they keep within the ordinary law of the land. No Secretary of State is required to defend, if he can, the acts of the committee; or, if he cannot, to take what is called suitable notice of them. Yet hospitals are a public necessity. Mr. Lloyd George cannot imagine any country allowing a single hospital to be closed; * and though people with stronger imaginations, or perhaps a closer acquaintance with some single hospitals, may hesitate to adopt his actual words, all must admit the conclusion intended to be conveyed.

It might be thought that any condition of affairs under which a public necessity is supplied by private generosity, would bring out some of human nature's better feelings. Unfortunately, the reverse is the case. Every hospital manager knows that the coroner's jury, the Poor-Law guardian, even newspaper editors of a kind, fall with great ease into a state of joyful readiness to fear the worst, whenever a hospital is attacked. It is far too late in the day to tell them that the subscribers can do as they please with their own. The responsibility of a hospital committee can no longer be limited to a roomful of empty chairs called the annual meeting of governors and members.

The chief importance of this fact, at the present time, lies in the danger of State interference with hospital work; and those who are, as I am, strongly opposed to any change in the voluntary system will do well to consider in advance all matters which relate to the task of disarming our opponents.

It will, I think, be admitted that destructive legislation is nearly always preceded by a cloud of prejudice; and in all attempts to deal with prejudice which I have had opportunities of observing, nothing has struck me more than the important part which responsibility plays. Indeed, responsibility, placed on the right shoulders, would have killed half the so-called hospital scandals of recent years, even after the fact; while, under a proper allocation of it from the outset, the other half would never have come to birth at all. For, in all relations of life, the sooner you place responsibility on the really responsible person who is causing trouble—whether it is

* *Times*, July 26, 1911.

responsibility of dying in prison from unnecessary hunger, or of dying outside from repletion—the sooner the root cause of the trouble will disappear.

In the case of hospitals, an example of divided responsibility, which at once occurs to the mind, is to be found in the work of the staff and the lay committee. About professional work there should be no question.* But there is a wide stretch of country between the physician's prescription and the kitchenmaid's evening out. Where is the line drawn? Is there not a belt of dangerous neutral territory on which we should expect to see any possible trouble with the public arise?

Let me suggest an answer by giving an instance. As chairman of a hospital I was once threatened with the newspapers in connection with a dissatisfied out-patient. A doubt was raised on our own side as to whether there was anything the matter with him; and, being only a humble syllogistic reasoner, I thought that was the first point to clear up. So I said that, if the complainant would come to the hospital, a general examination into his state of health should be made. But the doctor who was requested to make this examination only complied under protest. He said I had no business to put such work on the staff. Well, perhaps he was right; but if so, it was clearly hard on the committee that they should be brought into the matter, one way or the other. The public will attack a committee when it would hesitate to question the professional opinion of a doctor. Yet in the case in question, the doctor being satisfied himself (as I was myself) that the man was an impostor, but not being in any danger (as we were) of coming personally under the lash of an evening paper, was with difficulty persuaded not to leave the laymen to their fate.

Another example of responsibility divided between staff and committee is to be found in connection with the admission of patients—a frequent subject of complaint by hospital critics, at any rate in London; and I can only speak of London hospitals. It is said that wards are unduly filled with doctors' "request" cases, and that "uninteresting" illness is at a disadvantage.† I do not express any opinion on the merits of such criticism. But it is clear that no one but a doctor can say whether a doubtful case is a proper one for admission; and personally I believe that, in this matter, the public would, on the whole, be better off in the doctors' hands alone, than under any system of interference by the subscribers. A case came to my notice, last year, where a farm labourer met with a serious accident and was taken six miles across country to a large provincial hospital. He was, of course, admitted. But, afterwards, his wife and two small children were found wandering about the country trying to obtain an in-patient letter. It turned out that this had been demanded on the ground that, under the rules, the hospital was not bound to take in, but only to "treat" emergency cases, free. It is difficult to imagine the circumstances, short of extraordinary coincidence, under which this rule can ever be brought into operation, without either abuse of the charity, or inhumanity. But it is easy to see that, in any case, the doctors would be no party to its habitual enforcement, and equally easy to imagine how the existence of such a rule might be used as a stick with which to beat the voluntary hospital system. I do not think that it would ever have found its way into print if the doctors, who in cases of emergency are the really responsible persons, had settled the regulations for admission.

Internally, therefore, it seems to me to be of importance that, as far as possible, there should never be two kinds of responsibility attached to one class of hospital business, whether that business is medical, domestic, legal, financial, sanitary, or religious. The mind that conceives should belong to the body which will suffer if the conception gives offence.

Externally, the rise of the central collecting fund also raises some important considerations connected with responsibility.

The managers of such funds owe their first duty to those whose money they handle. They must consider destination, necessity, and economy. If a question arises as to money granted going directly, or indirectly, to medical schools, they will properly take the best advice they can get, the more independent the better, and act upon it.* If a hospital is already fully endowed, they will not proceed to over-endow it by grants. If a hospital wastes its subscribers' money, they will not give it more than would have been necessary had it acted prudently.

---

* There are, however, from time to time, cases where a committee, responsible for expenditure, refuses to carry out the orders of a single doctor. For example, a secretary writes to me: "A physician here ordered strawberries in March for a patient, at 7s. 6d. a pound. The patient was suffering from some obscure West African disease. I refused to supply. The physician insisted and took the matter to the committee. The committee supported me, and stated that they would take responsibility for their action." In such cases the position in which the committee are placed is always very unsatisfactory; and I do not myself see any half-way house between ceasing to admit certain classes of cases altogether, if funds will not permit, and leaving any disputed points about treatment to the final decision of the staff.

† From January 1 to August 31 of the present year a surgeon with 20 beds in one of the London hospitals sent in 74 request cases. In 1911, at the same hospital, the total of the request cases amounted to over ten per cent. of the total admissions. The request cases were admitted on sight, and, subject to their priority, ordinary cases were admitted in turn.

* I refer here only to the fundamental question—yes, or no, can the money granted be applied to a particular object? That being settled, primary responsibility for *misapplication* of any grant ought, in my view, to rest with the hospital treasurer who misapplies, remembering that he himself occupies a high position of trust, and, as I have said, is, in fact, responsible to the public. If he is charged, from outside, with misapplying his grant, the original grantor must then do justice by providing means, acceptable to all reasonable parties concerned, for an impartial inquiry, at which the accuser would have to make out his own case. Any other course is bound to end in trouble and more or less damaging criticism on the part of the general public, owing to a faulty distribution of the proper responsibility incurred by the various parties. I need scarcely add that I am not considering the improbable case of a lunatic or criminal treasurer, who, point blank and in open defiance, sends his grant bodily at an object he knows to be the wrong one.

In all these matters the fund must take responsibility for its own acts.

But, in order to be able to act, they must be in a position to judge. They cannot judge without uniform accounts and statistics from the various institutions; and an excellent example of the wise allocation of responsibility within responsibility is to be found in the course taken on the establishment of the existing revised system, which is the work of representative hospital secretaries themselves, assisted by the highest professional advice.

These, as far as I can see, cover the duties of a collecting fund to its subscribers. But there are some useful things it can do for the hospitals as well. It may disseminate information which only a central fund can conveniently collect, such as the statistical report of the King's Fund. It may arbitrate in cases of dispute, if invited to do so; and it can often obtain valuable assistance for work of that kind which would not be open to individual hospitals.

But anything beyond this should be very carefully considered. There may be no harm in sending visitors to make an amateur inspection of hospitals, though I think myself that, as a rule, visitors learn much more on their visits than they can teach. I am, however, very strongly opposed to a central authority giving any definite advice on internal management, unless it is prepared to take full responsibility for the consequences. That is a test which, in common fairness, should never be omitted. It is one thing to warn a hospital that if it builds a large new wing it must not look to any particular fund for an increase in its grant. It is quite another to demand plans of a building, whether admitted to be necessary or not, and then to discuss questions of construction. To do that is to leave the beaten path of finance and enter upon most dangerous ground; dangerous for the fund if a clear, decided opinion is given; paralysing and demoralising for the hospital managers if the views expressed by the holders of a long purse are halting, or hedged with provisos intended to make them safe in any event.

The very life of the voluntary-hospital system is local management. If that is once touched by central authority, in the matter of details, the best argument for retention of the voluntary system is taken away. With any such proceeding I will, in no case, be associated; but if others differ, at least I may venture to advise that responsibility shall never be divided. For, official advice accompanied by the giver's undivided responsibility has a tendency to shyness; but when it does face the footlights alone, it, at any rate, gives all the guarantee in its power.

I should not have thought this subject worthy of being brought before the British Hospitals Association—as so much of what there is to be said is self-evident to hospital managers—were it not that the outlook for the future, with all the signals set at danger, seems to me to make every point which may, even temporarily, be lost sight of, an important one. This subject of responsibility I regard as a pass key which ought not to be mislaid or allowed to rust, and which, as time goes on, will be found of increasing consequence. I have not attempted to do more than to stimulate discussion; and that, I take it, is the proper object of a conference paper.

### The Discussion.

The PRESIDENT then invited discussion, and called upon Mr. A. W. WEST, Chairman of the Council of the Association:—

I am, of course, only expressing a purely personal opinion when I say I think Mr. Danvers Power has struck the keynote of the whole voluntary system. Before any change is made in the present voluntary system of hospital management this question of responsibility is one which will have to be very carefully considered, and I most earnestly hope that the voluntary system will still be maintained. In using the expression "responsibility," Mr. Danvers Power truly points out that it is of a dual nature. In the case of the secretary who took upon himself not to carry out the order of the physician in regard to the strawberries, surely he was wrong in a double sense. The responsibility lay with the physician who ordered the strawberries. Who would have received the blame had the man died and it had come out in evidence at an inquest that part of the treatment ordered by the physician had been refused by the secretary? The hospital would have been blamed. It was therefore clearly a matter for their decision and not that of the secretary. I remember the case of a hospital in which it was considered that the amount of spirits consumed was excessive. Three monthly returns showing clearly the amount ordered by each physician and surgeon were made out and circulated among the staff. The amount of spirits ordered was reduced by one-half very quickly, because the responsibility was placed upon those ordering it to justify their action. In regard to Mr. Power's remarks as to the misapplication of any grants, that subject is one in which I feel a keen personal interest. I am glad to say that here again I am in agreement with the writer of the paper. If any accusation is made of the misapplication of any moneys, an impartial inquiry should be held and the accuser should be called upon to make good his case, or else trouble will ensue. That brings me to the further question of the system of hospital accounts as at present in general use. It is a subject to which I think the Council of the British Hospitals Association may well devote their attention in the coming year. The present system is no doubt a great improvement on previous methods, but by the light of experience it is very evident there is room for still greater improvement. The present system does not show a true comparative cost as between one hospital and another.

Colonel ROXBURGH, Chairman of the House Committee of the Western Infirmary, Glasgow:—

It seems to me that there is no getting away from the position that the committee managing a hospital are the responsible people for everything, whether from the administrative or the medical point of view. I do not mean to say that the managers are to interfere in the medical management of the hospital, but that they have to accept the ultimate responsibility I am quite convinced. In the hospital with which I have to do we have a board of twenty-seven managers who represent the community very largely. But we have what you have not very often in England, a medical superintendent; and although he is in no way responsible for the treatment of the patients, he acts as an excellent go-between between the managers and the medical staff. We have constituted six senior surgeons a consultative committee, and if anything arises which the managers do not quite see their way through, they refer it to the consultative committee. On the other hand, if the consultative committee have any suggestions to make to the managers, they have no hesitation in coming to us. In that way we get over any friction. I can quite understand that where there is a lay secretary of the hospital managing matters there might be some difficulty. Of course, the admission of patients is a matter which rests with the doctors. The committee will not interfere with them. We still work under the old system of "lines." In urgent cases the friends are asked if possible to get a "line." The doctor passes the case or not as he thinks fit. Of course, we all have a long waiting list, and we know that a number of those cases are not urgent, and that the urgent cases must have the preference. I am told that some people say that the "lines" are antiquated, but we hesitate about abandoning a system which gives subscriptions. The "line" bears distinctly the statement that the patient is to be admitted only if found suitable. In Scotland we have no difficulty with any fund on which we have any claim. We have no central fund which can give us large grants; the only thing we have is a hospital fund, which is distributed according to the number of patients treated. I never heard of anyone refusing a grant because they had too much money. (Laughter.)

Mr. Gilbert Barling, Senior Surgeon, General Hospital, Birmingham:—

I am practically the only representative of the staffs of the famous Birmingham hospitals, and I feel almost inclined to apologise for intruding into a meeting of this kind. ("No, no.") I agree that there must be one responsible authority for the administration of the hospital, and not more than one. The managing committee or board must be the authority. The question is, How can you satisfy the voluntary medical staff without splitting the authority? We have a very good system at the General Hospital. There is a managing board and the senior medical staff are members of the board. The medical staff has the sense to recognise that if they make demands on the hospital board that cannot be complied with, the board decides and the question is at an end until there is more money at their disposal. There is so much to be said for the voluntary-hospital system that I desire that it will continue to be our system. The question of efficiency turns largely on the question of money. At the present time we are asking our board to develop very largely the pathological side. We believe this is going to influence in the future the well-being of the patients. The surgeon will be required as far as we can see for a long time; the physician will also be required, but each will require more and more assistance from the scientific side of medicine. Hospital treatment is bound to be more and more expensive as it is more and more specialised. Yet one cannot help being struck with the way in which expenditure in various directions is kept within bounds by constant supervision of accounts. If you watch accounts you will frequently put your finger on a spot where there has been expenditure which is not justified. We know that we are exposed to criticism from every side. If the reporters had not been here I should have said that some of the papers seem to take a delight in girding at us. (Laughter.) But we are very much in the position of the countess who appears in the Divorce Court, or the clergyman who is supposed to have done something wrong. We are generally grouped with things of that kind. (Laughter.) A lay committee cannot say, "We won't supply certain things." The best plan is to supply them and then to send a reminder that so-and-so has been ordered, that it seems an extravagant expenditure, but leave it at that. You may depend that if one man orders strawberries at 7s. 6d., his colleagues will want to know why he is being so extravagant, as their patients would suffer in consequence of there being less money for them. Take the next point mentioned in the paper. I think he was absolutely right to ask a member of the staff to examine the patient, and I think the member of the staff should have done it cheerfully because he is bound to consider the best interests of the hospital. Now I come to another point, that is, the question of the "request" cases. How are cases to be admitted? There are various ways. First of all you can admit them without any restriction; secondly, you can insist on a ticket; and, thirdly, you can do the worst of all things, charge them a registration fee. (Laughter.) At the General Hospital our ticket system brings us in about £3,000 a year. Suppose we give it up, what is going to make up the loss? The best plan is to retain the ticket system but to work it in a reasonable spirit. To send the friends or relations of a sick person hunting round for a ticket in an urgent case is a gross abuse of the system. (Hear, hear.) What right has an individual physician to say: "That is a suitable case and I send it in. I ignore the regulations?" I go down to the hospital on Thursday mornings to see people sent up for consultation. I see a man and I say to him, "You ought to come in. I think you have a very bad ulcer of the stomach which needs operation, but you will get quite well after many years of ill-health." I send that patient in without a ticket and often prior to men who have been waiting some time. I am prepared to defend that system to the utmost. I endeavour not to take any cases that can reasonably get in-patient tickets, but that is only a limited number. Most of them are cases that ought not to be harried about for tickets. As to money granted in support of medical schools, I think the more completely separate the two things are kept the better. Let them both stand on their own feet. But I would have no carping criticism. I think in London there has been carping criticism, and I don't think a great deal of it has been just. To many hospitals the pathological service is intimately connected with the teaching of students, but here, I am glad to say, the two things are practically distinct. It is easy to say, "Why don't you copy France, or Germany, or Australia?" You cannot. You cannot take the hospital system of one country and graft it into the other. Let the responsibility be with the hospital management board, but as far as possible satisfy every interest.

The Rev. M. Campbell, Chairman of the Dumfries and Galloway Royal Infirmary:—

Like Colonel Roxburgh I am not in a position to offer any opinion whatever on the second part of the paper—that referring to external management, because, unhappily, at my infirmary we have to deal with no outside body: all that is raised has to be raised by the managers themselves, we have no Hospital Saturday or Sunday Funds. The question of internal responsibility I think is one of the very utmost importance. As to the question of the admission of patients it has been claimed that this lies entirely in the power of the medical and surgical staff. I venture to disagree. I think the directors have also a very important interest in the matter. My committee claim (and they exercise it) that once a week the house committee should revise the list of admissions. That is important because people may be admitted who ought not to be admitted. There are cases which come under the Poor Law. In the case of the strawberries which has been mentioned I quite agree that that was a matter for which the medical staff was responsible. Then, again, there is no finality in the matter of hospital administration. In a place like a hospital you may have rules and regulations, and you ought to have them, but you will never cover the ground by the most complicated series of rules which you can devise. In the absence of a medical superintendent I am sorry to say the burden of reconciling conflicting interests very often falls on the chairman. If the chairman is sensible he will not carry everything to his committee.

Mr. Montague Browne, of the Northampton General Hospital:—

The question of divided responsibility should be faced more and more, and the mutuality of the responsibility more and more recognised. Our subscribers are the persons who have most of the responsibility thrust upon them, and they are the persons of all others who seem to me the least to recognise it. I speak from a very long experience, but I have never known a subscriber who has wantonly done a mean or shabby thing, but I have known many who have not faced the responsibility which is involved in holding that valuable piece of paper called a "letter," one of the most extraordinary things ever invented for afflicting mankind. Patients do not always recognise their responsibility. Every one of our patients who leaves our building has to sign a paper to say that he has been well treated. One out of so many hundreds afterwards finds when he gets home that he has been rather badly treated. (Laughter.) We then have to follow up those cases, but, of course, most of them can easily be disposed of. We have been told over and over again to-day and yesterday, "It is your money that we want." I venture to say that you can get money too dear. You can buy gold too dear. One day a question came up about collecting for the hospital. My matron told me that the nurses had told her that they would not collect in the street in future. I said, "I am very thankful to hear that." It may be a fad, but I cannot see that we want to get money at the expense of women standing or sitting in the public streets jangling boxes in the faces of men they have never seen before.

Mr. Conway Lowe, Birmingham and Midland Eye Hospital:—

With reference to the remarks made as to the medical treatment of patients who are too well off to be hospital patients. In 1909 four private wards were opened at the hospital and 187 patients have passed through. Whether this would be applicable to larger hospitals I cannot say, but we find it works smoothly. The charge is at the rate of £2 2s. a week, but the patient is required to make his own arrangements with the surgeon as to his fee. The wards are almost continually full, and experience shows that they are self-supporting.

Mr. T. Maddock, Wellingborough Cottage Hospital:—

As honorary secretary, I am called upon to give decisions in various matters, and when occasion arises for it I invariably throw the responsibility on the chairman. If I had been called upon to decide with regard to 7s. 6d. strawberries, I should have said, "No, we cannot." If it is a difficult matter, let the medical man decide it with the committee later on.

Mr. Kay, Chairman of the House Committee of Bolton Infirmary:—

I am sorry that no reference has been made to the work of ladies. We have six ladies on our committee, and two retire every year. If it is good to have ladies on the house committee at Bolton, it is good to have them in other places. I think we ought to get the Government to acknowledge us and to give us a grant every year, the same as they give a grant to policemen.

Dr. Cureton, Hon. Physician, Salop Infirmary:—

I want to emphasise the remarks of Dr. Barling with regard to the relationship that should always subsist between the board of directors and the medical staff. The visiting physician and surgeon for each week is an ex-officio member of the board of directors, so that the work done by the board of directors each week becomes the public knowledge of the medical board. Further, in order to get the full use of the knowledge of the medical board, we have a combined board consisting of board directors and the medical staff. That body elects the house surgeon, secretary, and so on. The medical board select suitable candidates from the various applicants, and they are brought before the combined board. With regard to the ticket system, I should like to say that some time ago an attempt was made at our infirmary to do away with subscribers' tickets. I consider, and the subscribers consider, it a privilege to be connected with a charitable institution, and they find a considerable amount of interest in giving out subscribers' tickets. With regard to the prescriptions of medical men, they are often very expensive. Not long ago we had a junior physician who ordered something which cost 15s. a day, but it was allowed and referred back to the medical board. With regard to the position sanatoria will occupy under the new Act, we have in Shropshire a small sanatorium, and on July 15 we came under the Act. Within a fortnight the Local Government Board sent an inspector down to look over it and the system by means of which we admitted our patients. I am one of the referees. He disagreed with my books, and said we were to adopt the card system, and so on. I don't know whether other hospitals will be subjected to the same inspection, but I think we ought to be careful about allowing them, because we shall find, if we don't mind, that we are taken over.

Mr. Howard J. Collins, General Hospital, Birmingham:—

It seems to me that the two instances which Mr. Danvers Power has given in his paper on the question of what might be considered to be difficulties of management are simply questions of the reasonable interpretation of rules. One speaker says rules will not cover everything. That is one of our reasons for having officers. For all practical purposes it makes no difference, to my mind, if the officer concerned has a reasonable knowledge of the working of a hospital. Both in the strawberry case and in the case of the unfortunate man who met with the accident the rule was probably absolutely correct, but the interpretation of it was exceedingly feeble. In the one case the strawberries should have been supplied, but in our case we should have mentioned it to the physician and then referred to the medical board. In the case of the ticket, undoubtedly a great harm would come if people were badgered to get tickets. Twenty-five per cent. of our in-patients only bring tickets, and 22 per cent. of the out-patients. Everyone who does not come as the result of an accident is asked to obtain a ticket. That is our means of livelihood. In no case is a patient ever refused for want of a ticket, or made to suffer because he has not got one, but no one in the hospital knows whether they have or not. I am sure every board is the responsible party, and the officers are only the representatives of that body, and it is their business to find out what the facts are and allow the board or managing committee to decide what is to be done in future.

Mr. Shaw, Secretary, Southport Infirmary, expressed his regret that the Council had not sent copies of the papers in advance to members of the Association. He thought it would very greatly facilitate the discussion. As regards the paper, I must say that personally I have derived very great advantage from the remarks of Dr. Barling, the senior surgeon of the General Hospital. I am quite in accord with what has been stated, that there should be no two kinds of responsibility in regard to hospital management.

Mr. West said the question of circulating the papers before the Conference would cut both ways. It would be interesting to receive them beforehand and think them over quietly, but how many people would come to the Conference?

Mr. Collins: They are not all members who are here.

Mr. J. E. Smith, Royal Hospital for Children, Bristol:—

It has been said that we come here to hear papers read. I very much doubt that we hear them read, but if we only wanted to know what was contained in the papers we should wait until the Conference has met and buy a copy of The Hospital. There is a great deal of good which the Conference does in discussions of hospital matters quite out of school. Our hospital has been conducted on the principle of no votes. It has been affirmed that if you did not give votes you would not get money from subscribers. That is not so. Our Children's Hospital at Bristol is one of four of the great local charities. I think we are the only one of the three where they issue no votes. Do people tell us they won't subscribe on that account? It is not so.

Mr. Danvers Power, replying on the discussion, said one of the most important factors of the whole consideration was the proper location of the responsibility in respect of the points as to which the public was inclined to make mischief. Dr. Barling's speech was precisely the sort of speech you like to hear from a man who has given his life to matters connected with his own profession, and who comes occasionally to a meeting of that kind to give his opinion, but unfortunately these were not the days when opinions of men of that kind would be accepted by party politicians. He (Mr. Power) was very much afraid they would find in other parts of the country, if not in Birmingham, that the system of State hospitals or municipal hospitals would provide very inferior results to the great ones now being shown by the present voluntary system. He was amused to find Dr. Barling pinning his faith to a municipal system of hospitals. That was precisely what they would expect to hear from Birmingham, which had set an example to the country.

Votes of thanks to the Lord Mayor for the use of the Council Chamber, and to Mr. J. B. Clarke for his presiding, were carried with acclamation.

In reply, the President said he was struck with the extreme usefulness of the Association. The practical lesson of the Conference was, he thought, to watch the working of hospitals on the Continent.

